# Multi-HLA class II tetramer analyses of citrulline-reactive T cells and early treatment response in rheumatoid arthritis

**DOI:** 10.1186/s12865-020-00357-w

**Published:** 2020-05-18

**Authors:** Christina Gerstner, Sara Turcinov, Aase H. Hensvold, Karine Chemin, Hannes Uchtenhagen, Tamara H. Ramwadhdoebe, Anatoly Dubnovitsky, Genadiy Kozhukh, Lars Rönnblom, William W. Kwok, Adnane Achour, Anca I. Catrina, Lisa G. M. van Baarsen, Vivianne Malmström

**Affiliations:** 1 Division of Rheumatology, Department of Medicine Solna, Center for Molecular Medicine, Karolinska University Hospital and Karolinska Institutet, Stockholm, Sweden; 2 Translational Research Program, BRI at Virginia Mason, Seattle, (WA) USA; 3 Department of Clinical Immunology and Rheumatology and Department of Experimental Immunology, Amsterdam UMC, University of Amsterdam, Amsterdam Infection & Immunity Institute, Amsterdam, Netherlands; 4 Amsterdam Rheumatology & Immunology Center (ARC), Academic Medical Center, Amsterdam, Netherlands; 5 Department of Medical Sciences, Rheumatology, Science for Life Laboratory, Uppsala, Sweden; 6 Science for Life Laboratory, Department of Medicine Solna, Karolinska Institutet & Division of Infectious Diseases, Karolinska University Hospital, Stockholm, Sweden

**Keywords:** Autoimmune disease, Autoreactive CD4+ T lymphocytes, Multi-MHC class II tetramer assay, Citrullination

## Abstract

**Background:**

HLA class II tetramers can be used for ex vivo enumeration and phenotypic characterisation of antigen-specific CD4+ T cells. They are increasingly applied in settings like allergy, vaccination and autoimmune diseases. Rheumatoid arthritis (RA) is a chronic autoimmune disorder for which many autoantigens have been described.

**Results:**

Using multi-parameter flow cytometry, we developed a multi-HLA class II tetramer approach to simultaneously study several antigen specificities in RA patient samples. We focused on previously described citrullinated HLA-DRB1*04:01-restricted T cell epitopes from α-enolase, fibrinogen-β, vimentin as well as cartilage intermediate layer protein (CILP). First, we examined inter-assay variability and the sensitivity of the assay in peripheral blood from healthy donors (*n* = 7). Next, we confirmed the robustness and sensitivity in a cohort of RA patients with repeat blood draws (*n* = 14). We then applied our method in two different settings. We assessed lymphoid tissue from seropositive arthralgia (*n* = 5) and early RA patients (*n* = 5) and could demonstrate autoreactive T cells in individuals at risk of developing RA. Lastly, we studied peripheral blood from early RA patients (*n* = 10) and found that the group of patients achieving minimum disease activity (DAS28 < 2.6) at 6 months follow-up displayed a decrease in the frequency of citrulline-specific T cells.

**Conclusions:**

Our study demonstrates the development of a sensitive tetramer panel allowing simultaneous characterisation of antigen-specific T cells in ex vivo patient samples including RA ‘at risk’ subjects. This multi-tetramer approach can be useful for longitudinal immune-monitoring in any disease with known HLA-restriction element and several candidate antigens.

## Background

HLA class II tetramers allow direct ex vivo detection and phenotypic characterisation of antigen-specific T cells [1] and are increasingly used in a range of different settings, like allergy, vaccination and autoimmune diseases [2–6]. This technology requires knowledge of both the antigenic peptide as well as the HLA restriction element, and hereby is utilised in conditions with known antigenic targets and a strong HLA component.

Rheumatoid arthritis (RA) is a chronic inflammatory disease and in a majority characterised by the presence of so called anti-citrullinated protein antibodies (ACPA). It is highly associated with a certain set of HLA-DR alleles [7–9] and a number of citrullinated T cell targets have recently been identified [5, 10–14]. To detect rare antigen-specific CD4+ T cells ex vivo, others and we made use of a protocol first published by Wucherpfennig and colleagues [15] that combines tetramer staining with magnetic bead enrichment. By this means, we previously quantified and phenotypically characterised T cells specific for epitopes from several RA-associated candidate antigens in a variety of different RA samples, including peripheral blood mononuclear cells (PBMC), synovial mononuclear cells and synovial tissue [5, 6]. However, a major shortcoming of this method is the need for large samples, where the study of one epitope typically requires 20–30 million PBMC.

Based on the combinatorial HLA class II tetramer staining approach demonstrated for immunodominant viral epitopes by Uchtenhagen et al [16], we thus set out to develop a multi-tetramer assay for detection of autoreactive T cells that would render it possible to investigate numerous specificities simultaneously and in parallel retain high sensitivity allowing identification of rare autoreactive CD4+ T cells. We here focused on known HLA-DRB1*04:01-restricted citrullinated T cell epitopes from some of the most common RA-associated autoantigens, namely α-enolase, vimentin, fibrinogen beta chain (FGB) as well as cartilage intermediate layer protein (CILP) [5, 14].

ACPAs have been shown to be present in individuals developing RA decades before onset of disease [17]. Moreover, studies on a large twin cohort examining genetic and environmental factors in the development of RA suggested that specific HLA class II alleles and thus probably CD4+ T cells are likely to be involved in the maturation of the ACPA response, i.e. epitope spreading shortly before disease onset, and the subsequent initiation of arthritis [18]. However, studies on autoreactive CD4+ T cells ex vivo prior to or at disease onset are currently not available in RA and the role of autoreactive CD4+ T cells in the pathogenesis of RA needs to be further investigated. We therefore studied the autoreactive T cell profile, specificity, phenotype and frequency, in individuals with seropositive arthralgia who are at risk of developing RA, and in early, untreated RA patients at disease onset. To this end we screened lymph node (LN) core needle biopsies from arthralgia patients and compared them with early, untreated RA patients. Next we investigated PBMC collected from early RA patients that were sampled prior to treatment initiation and at 6 months follow-up visit [19].

Altogether, this study demonstrates that a detailed analysis of citrulline-specific T cells using a multi-tetramer assay is feasible in both individuals at risk of developing RA and patients at disease onset. Even though at low frequencies, these autoreactive T cells mainly displayed a memory phenotype at time of RA onset. Moreover, certain autoreactive T cells were found to decrease in frequency in patients achieving minimum disease activity following 6 months treatment. Hereby, we could substantiate the utility of this approach for future longitudinal immune-monitoring.

## Results

### The multi-tetramer approach allows stable detection of autoreactive T cells at low frequencies in healthy controls

**Fig. 1:**
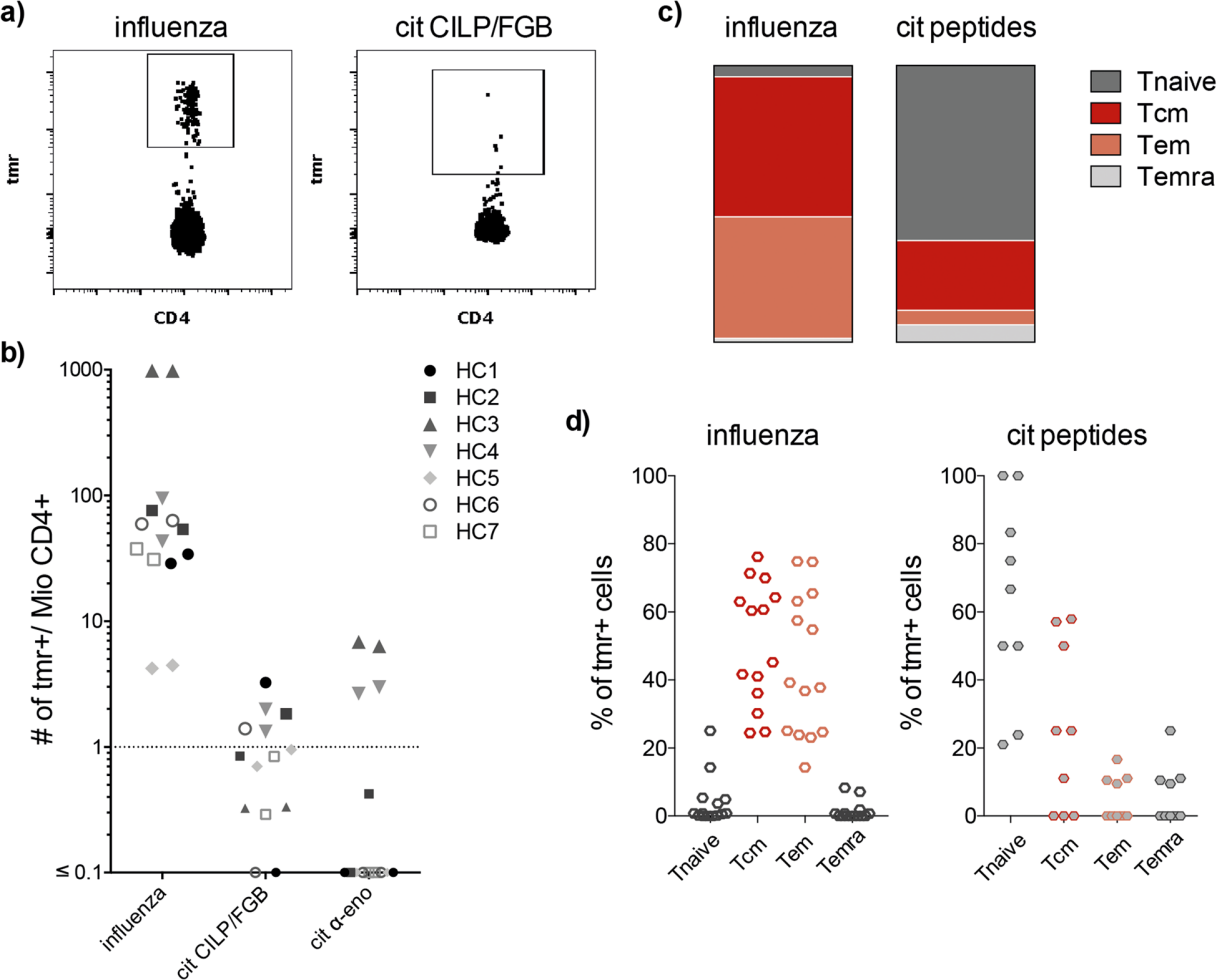
The multi-tetramer approach is sensitive enough to detect antigen-specific CD4+ T cells in healthy controls. **a** Representative flow plots depicting the gating strategy for CD4+ T cells reactive to influenza (left) and citrullinated CILP/FGB peptides (right). **b** Frequency of antigen-specific CD4+ T cells is shown for seven healthy controls (different symbols and shades of grey for each buffy coat). Plotted are tetramer-positive cells per million CD4+ T cells from all fourteen experiments (one technical replicate per healthy control) for influenza, citrullinated CILP/FGB and citrullinated α-enolase. Cut-off for positivity is one tetramer-positive cell per million CD4+ T cells, marked with a dotted line. **c + d** Characterisation of antigen-specific CD4+ T cells by differentiation status, determined by simultaneous or singular expression of CD45RA and CCR7 according to Sallusto et al [22] in naïve (Tnaïve), central memory (Tcm, coloured in red), effector memory (Tem, coloured in salmon) and CD45RA+ effector memory (Temra) T cells. We plotted the proportion of influenza- and citrulline-specific T cells among the four different phenotypes in **(c)** box plots showing the mean distribution and **(d)** scatter plots showing the detailed proportion and distribution of influenza- (left, open symbols) and citrulline-specific (right, closed symbols) T cells among the different phenotypes

To study citrulline-reactive T cell specificities with our multi-tetramer assay, we created a panel of HLA-DRB1*04:01 tetramers loaded with eight citrullinated RA-associated self peptides derived from four candidate autoantigens, namely α-enolase, vimentin, fibrinogen-β as well as CILP. Additionally, we employed two commonly used viral control epitopes from influenza matrix proteins [20, 21]. Table 1 lists the sequence and position of the peptides as well as the tetramer fluorophores. As autoreactive T cells have been shown to be present at low frequencies in the circulation of RA patients and healthy individuals [5, 6] we first determined the sensitivity of our multi-tetramer assay by applying it to PBMC from seven HLA-DRB1*04:01-positive healthy control subjects. Here, we unambiguously detected T cells specific for α-enolase, CILP and fibrinogen at frequencies ranging up to 10 per million CD4+ T cells (median frequency 4.7 and 1.7 per million CD4+ T cells, respectively; Fig. 1a and b). As expected, the number of cells reactive to our positive influenza peptide controls was higher, typically in a range of 20 to 1000 per million CD4+ T cells (median frequency 61 per million CD4+ T cells). Repeat experiments performed on cells from the same time points and donors demonstrated on average comparable frequencies of tetramer-positive cells in the two experiments for each donor (Fig. 1b).

**Table 1:** Epitopes used in this study

Name	Protein	Sequence	Fluorochrome	Reference	
cit-eno 11–25	α-enolase	11-IFDSXGNPTVEVDLF	PE	5	
cit-eno 26–40	α-enolase	26-TSKGLFXAAVPSGAS		14	
cit-eno 326–340	α-enolase	326-KXIAKAVNEKSCNCL		5	
cit-CILP 297–311	CILP	297-ATIKAEFVXAETPYM	PE-CF594	5	
cit-CILP 982–979	CILP	982-GKLYGIXDVXSTRDR		5	
cit-fibβ 69–80	fibrinogen β	69-GYXAXPAKAAAT		5	
cit-vim 59–78	vimentin	59-GVYATXSSAVXLXSSVPGVR	APC	11	included in LURA and LN experiments
cit-vim 418–431	vimentin	418-FSSLNLXETNLDSL		5	
HA 306–318	influenza HA	306-PKYVKQNTLKLAT	PE-Cy5	20	excluded in LN experiments
MP 97–116	influenza MP	97-VKLYRKLKREITFHGAKEIS		21	

Abbreviations: *X* citrulline, *CILP* cartilage intermediate layer protein, *HA* hemagglutinin, *MP* matrix protein

Besides enumerating the tetramer-positive CD4+ T cells, we also determined their differentiation state by examining the surface expression of CD45RA and CCR7 (Fig. 1c and d and Additional file 1: Figure S2a). As expected, T cells specific for influenza were predominantly of a memory phenotype and distributed between a Tcm, central memory (51%) and a Tem, effector memory phenotype (44%). Conversely, the majority of autoreactive T cells in these healthy subjects displayed a naïve phenotype, expressing CCR7 and CD45RA simultaneously (Fig. 1c and d). Still, it should be noted that we also detected central memory type T cells in a subset of the samples, while effector memory T cells were consistently a minor phenotype.

### Autoreactive T cells are found in most RA patients, even in the absence of concurrent disease activity

Next, in order to further validate our panel also in patient samples, we analysed a longitudinal cohort of 14 RA patients from which we obtained samples from repeat blood draws approximately 2–3 weeks apart and therefore could analyse intra-individual variance. The patients included in this cohort were recruited according to the following criteria: having ACPA-positive RA and at least one HLA-DRB1*04:01 allele. All patients had long disease duration (> 5 years), overall no signs of active disease around the time of sampling and stable anti-rheumatic treatment according to standards (see Additional file 1: Table S1.1).

**Fig. 2:**
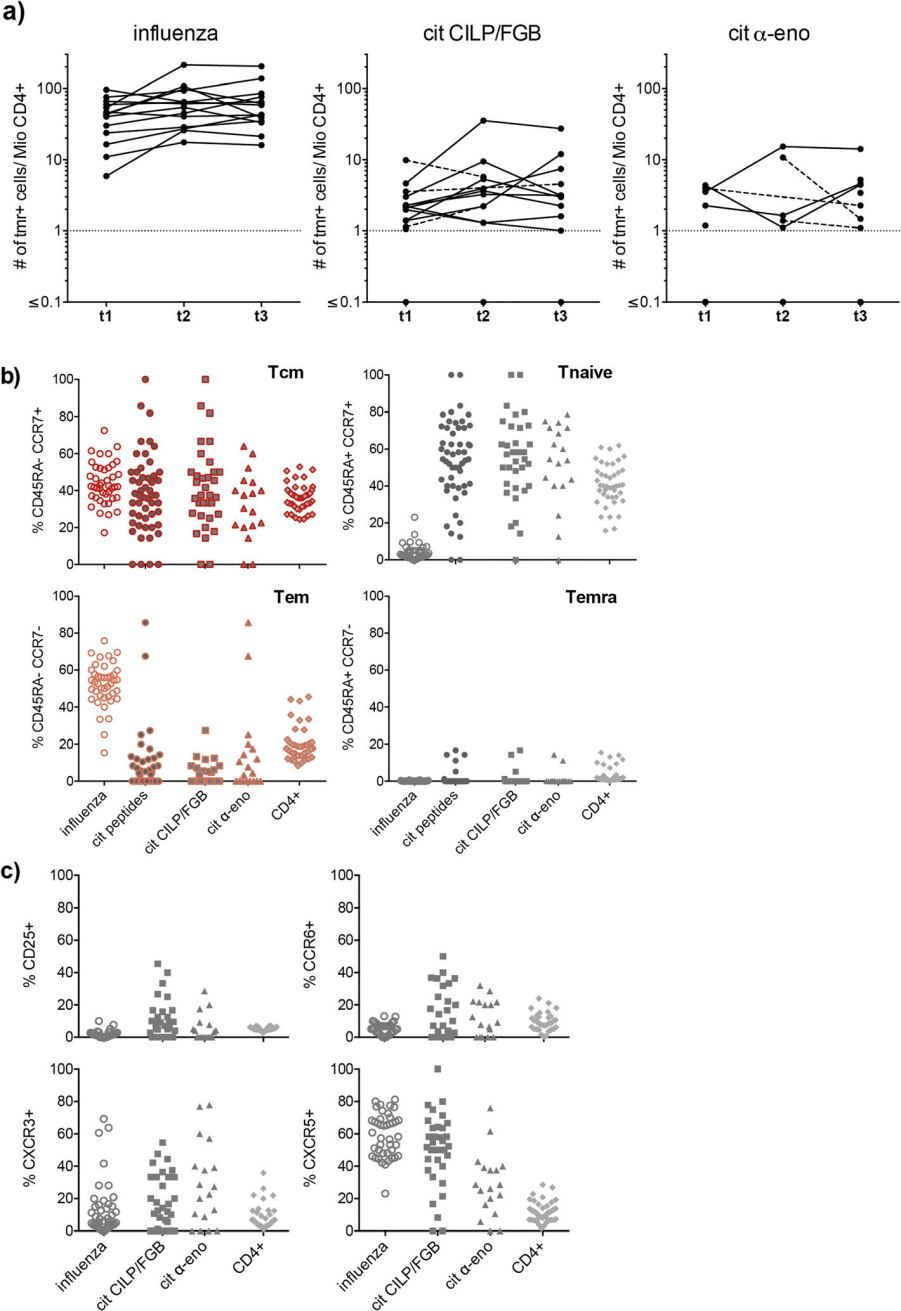
Autoreactive T cells in a cross-sectional cohort of RA patients. **a** Frequency of antigen-specific CD4+ T cells, recognising influenza, citrullinated CILP/FGB or citrullinated α-enolase peptides, is shown for all 14 patients longitudinally. Plotted are tetramer-positive cells per million CD4+ T cells. Cut-off for positivity is one tetramer-positive cell per million CD4+ T cells and marked with a dotted line. The frequencies detected at the three time points (t1, t2, t3) are connected with a continuous line for each patient and with a dotted line in case there were no cells detected at one or two out of the three time points. **b + c)** Phenotypic characterisation of antigen-specific CD4+ T cells according to **(b)** simultaneous or singular expression of CD45RA and CCR7 and **(c)** expression of CD25, CXCR3, CXCR5 and CCR6. Plotted are frequencies from all 42 experiments (three per patient) for influenza- (open circles) and citrulline-specific (closed circles, not included in c) as well as for CILP/FGB- (squares) and α-enolase- (triangles) specific T cells and the general CD4+ population (light grey diamonds). Due to the setup of the experiment which resulted in there being up to three individual data points per patient plotted for each of the different antigen-specific T cell populations, we did not perform any specific statistical analysis

We detected frequencies between 1 and 35 tetramer-positive cells per million CD4+ cells of CILP/fibrinogen- and α-enolase-specific T cells in these RA patients (Fig. 2a). These frequencies were slightly increased in patients compared to healthy controls (Fig. 1b and 2a). Not all specificities were present in all patients, with α-enolase-specific T cells being detected in eight out of fourteen and CILP/fibrinogen-specific T cells in thirteen out of fourteen patients. Specificities within individual patients were reliably detected in the repeat blood draws in half of the individuals. Other patients showed citrulline-specific T cells only at one or two of the three time points, as indicated by single dots and dotted lines connecting the frequencies of the remaining time points in Fig. 2a. In contrast, influenza-specific T cells were steadily found in all patients in each of the three repeats and always at 10–20 times higher frequency compared to autoreactive T cells (Fig. 2a). Examining the overall distribution of the cells within the different memory and naïve states, we detected - similarly to healthy subjects - a high proportion of influenza-specific T cells in the central and effector memory compartment and very little amounts of naïve T cells (Fig. 2b and Additional file 1: Figure S2b). Again, we found a broad distribution of the proportion of naïve citrulline-reactive T cells between different subjects. Within the memory subset, central memory type T cells were overrepresented among CILP/fibrinogen- compared to α-enolase-reactive T cells (Fig. 2b and Additional file 1: Figure S2b). To a lower extent (< 20%) we also detected citrulline-specific T cells with a terminal effector memory phenotype, such Temra cells were virtually absent amongst the influenza-specific T cells (Fig. 2b and Additional file 1: Figure S2b).

Analysing the expression of chemokine receptors on the tetramer-positive cells revealed a higher percentage of CXCR3+ cells among α-enolase- and CILP/FGB-specific compared to influenza-specific T cells (Fig. 2c). CXCR5 expressing cells on the other hand were mainly detected among CILP/fibrinogen- and influenza-specific T cells and rather less among cells recognizing α-enolase. We found little expression of both CD25 and CCR6 on the influenza-specific as well as the general CD4 population, while such phenotypes were detected in α-enolase- and CILP/fibrinogen-specific T cells (Fig. 2c). Of note, among influenza- and citrulline-specific cells as well as in the general CD4 population around half of the CXCR3+ cells also expressed CXCR5 and to a lesser extent CCR6 (data not shown).

### Citrulline-specific T cells are present in lymph node biopsies of arthralgia and early RA patients

Next, we applied the multi-tetramer assay to lymph node biopsies from HLA-DRB1*04:01-positive individuals. Included in this part of the study were arthralgia patients with elevated rheumatoid factor (RF) and ACPA levels as well as early RA patients. Samples from one patient with undifferentiated arthritis and one healthy control were also analysed (see Additional file 1: Table S1.2).

**Fig. 3:**
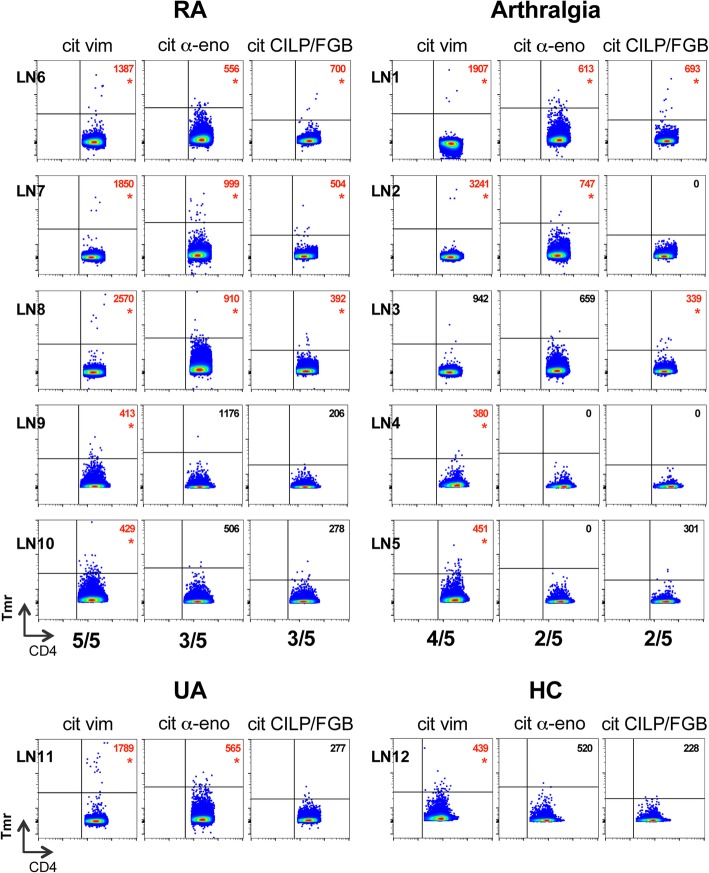
Citrulline-specific CD4+ T cells in LN biopsies from arthralgia and early RA patients. Lymph node biopsies were stained for citrullinated vimentin-, citrullinated α-enolase- and citrullinated CILP/FGB-reactive T cells after in vitro propagation using the tetramers according to Table 2, i.e. two cit vim-, three cit α-eno-, three cit CILP/FGB-peptide-loaded tetramers. Displayed are plots from both RA and arthralgia patients (upper panel) and one patient with undifferentiated arthritis (UA) as well as one healthy control (HC, lower panel) showing tetramer-positive cells against CD4. Numbers in the upper right quadrant present the mean fluorescence intensity (MFI) of the tetramer-positive staining, which can help in assessing positive staining. Cut-off for positivity for the different tetramer-cocktails are MFI > 400 for cit vim, MFI > 600 for cit α-eno and MFI > 320 for cit CILP/FGB. Plots marked with a red asterisk show positive staining. The numbers of positive tested individuals/tested individuals in total is depicted below the plots for each specificity for both RA and arthralgia patients

Due to the limited cell numbers that can be retrieved from a core needle biopsy we decided against the general ex vivo protocol but opted instead for analysing cells after in vitro expansion using phytohemagglutinin (PHA) and interleukin-2 (IL-2). We could detect citrulline-reactive T cells in all of the tested individuals albeit with varying number of specificities (Fig. 3). All five RA patients had vimentin-specific T cells while α-enolase- and CILP/fibrinogen-specific T cells were found in three RA patients. One of the five arthralgia patients displayed reactivity against all citrullinated peptides, while another one had vimentin- and α-enolase-specific T cells. The remaining three arthralgia patients had one of the three citrullinated specificities; two displayed vimentin-reactive and one CILP/fibrinogen-specific T cells. Also for the healthy control we could demonstrate the presence of vimentin-specific T cells, however no CILP/fibrinogen- or α-enolase-reactive ones. The patient diagnosed with undifferentiated arthritis had both vimentin- and α-enolase-specific T cells. It should be noted that since these cells required cell culture expansion, we could not calculate the original frequencies nor analyse the unmanipulated phenotypes.

### Citrulline-specific T cells decline in early RA patients achieving drug-induced remission

Finally, we applied the multi-tetramer assay to peripheral blood samples from ten patients enrolled in the lung investigation in newly diagnosed RA (LURA) study at the Karolinska University Hospital. This cohort comprises 134 newly diagnosed patients with duration of patient-reported symptoms ≤1 year and naïve to treatment with disease-modifying anti-rheumatic drugs (DMARDs) and oral glucocorticoids (GC). The selection of the patients for this substudy was based on HLA-DRB1*04:01-positivity and the availability of cryopreserved blood samples from both baseline and 6 months follow-up.

**Fig. 4:**
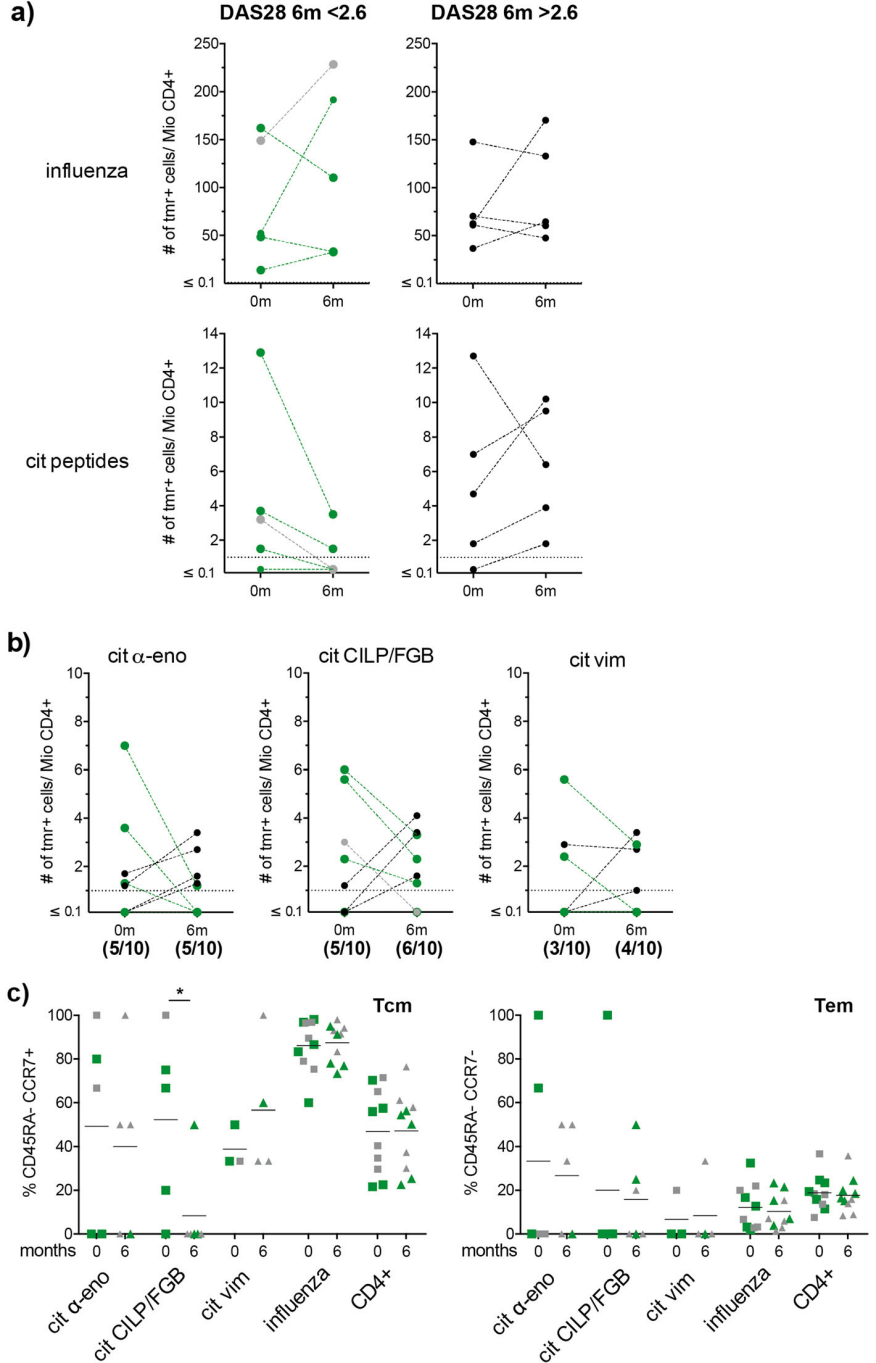
Early RA patients achieving drug-induced remission show a reduction in citrulline-specific T cells. **a + b**) Frequency of (**a**) influenza- and citrulline-specific T cells in patients achieving remission (DAS28 at 6 m < 2.6) versus patients not achieving remission (DAS28 at 6 m > 2.6) and (**b**) of T cells reactive to citrullinated α-enolase, citrullinated CILP/FGB and citrullinated vimentin is depicted at baseline (0 m) and follow-up visit (6 m) for 10 early RA patients. Plotted are tetramer-positive cells per million CD4+ T cells. Cut-off for positivity is one tetramer-positive cell per million CD4+ T cells and depicted by a dotted line. Green symbols and lines depict the patients that achieved clinical remission at follow-up. The patient with missing DAS28-value at 6 m is coloured in grey. The numbers of positive tested individuals/totally tested individuals for each time point is depicted below the graphs showing α-enolase-, CILP/FGB- and vimentin-specific T cells. (**c**) Phenotypic characterisation of antigen-specific CD4+ T cells according to simultaneous or singular expression of CD45RA and CCR7 in central memory (Tcm) and effector memory (Tem) T cells. Plotted are frequencies and the mean for citrullinated α-enolase-, citrullinated CILP/FGB-, citrullinated vimentin- and influenza-specific T cells as well as the general CD4+ population at 0 m (squares) and 6 m follow-up (triangles). Green symbols depict the patients that achieved clinical remission at follow-up. Statistical analysis was performed using Mann-Whitney test when comparing the frequency of central memory T cells at baseline with the follow-up time point. *p*-values less than 0.05 were considered significant and marked with an asterisk

T cells specific for both influenza and citrullinated peptides were found to be part of the peripheral T cell repertoire at both baseline and following 6 months of standard medication with methotrexate in this early RA cohort (Fig. 4a). Citrulline-specific T cells were detected in all but one patient, who interestingly turned out to be negative for both RF as well as ACPAs. The specificity that was detected least often was towards vimentin, with 3/10 and 4/10 positive patients for baseline and follow-up, respectively. Both α-enolase- and CILP/fibrinogen-specific cells were found in 50–60% of the patients at both time points (Fig. 4b). The general frequencies determined for influenza-specific as well as autoreactive T cells were in line with previous results and ranged from 10 to 230 and 1 to 7 tetramer-positive cells per million CD4+ cells, respectively. Notably, three of the four patients for whom the frequency of citrulline-specific T cells declined from baseline to follow-up, were also patients that achieved a disease activity score (DAS28)-value < 2.6 which corresponds to minimal disease activity and clinical remission (Fig. 4a, coloured in green and Table 2). Another patient could, due to a missing DAS28-value at follow-up, not be added to the group of responders, although we could demonstrate a decrease in citrulline-reactive T cells also for this patient (Fig. 4, coloured in grey and Table 2).

**Table 2:** Characteristics of selected newly diagnosed early RA patients

Subject	Antibody status (RF/ACPA)	DAS28 0 m	DAS28 6 m	Medication after baseline sampling
1	+/+	3.89	3.88	Mtx + GC
2	+/+	4.68	2.58^B, D^	Mtx + GC
3	+/+	5.04	1.05^B, D^	Mtx + GC
4	+/+	5.70	3.44^C^	Mtx
5	+/+	7.39	2.24/2.53^B, E^	Mtx + GC
6	+/+	5.47	3.46^C^	Mtx
7	−/+	4.90	2.84^A, B^	Mtx
8	−/−	5.52	2.39^B, D^	Mtx + GC
9	+/+	5.49	1.63^B, D^	Mtx + GC
10	+/+	4.31	2.63^A. B^	Mtx

Abbreviations: *DAS28* disease activity score 28, *Mtx* methotrexate, *GC* glucocorticoids

^**A**^ low disease activity; ^**B**^ remission; ^**C**^ moderate improvement; ^**D**^ good improvement; ^**E**^ DAS28 at 6 m not available, displayed are DAS28 at 3 m/13 m

Subsequent analysis of CD45RA and CCR7 expression revealed a significant decrease of central memory type T cells after 6 months amongst CILP/fibrinogen-specific cells. Along the same line we detected a general trend towards lower proportions of memory type T cells for both α-enolase- and CILP/fibrinogen-specific cells and concurrently higher proportions of naïve as well as terminal effector memory T cell phenotypes (Fig. 4c and Additional file 1: Figures S2c, right panel, and S3a). Conversely, vimentin-specific T cells displayed a trend for increased central memory and decreased naïve T cells at 6 months follow-up (Fig. 4c and Additional file 1: Figure S2c, right panel, and Figure S3a).

To investigate the activation status of these cells in more detail we analysed expression of CD25 and CD38, markers of recent T cell activation and detected no significant changes upon comparing baseline and follow-up for neither of the two markers (see Additional file 1: Figure S3b). Also, as expected for patients not subject to ongoing infections or recent vaccination, the level of expression on the influenza-specific control as well as the general CD4+ population was very low for both these markers.

Similarly, we examined the surface expression of the chemokine receptors CCR6, CXCR3 and CXCR5 and found a general decrease in the frequency of CXCR5+ cells for all autoreactive as well as the influenza-specific T cells at follow-up. In general, CXCR5, compared to CXCR3 and CCR6, is the chemokine receptor that was most prominently expressed on tetramer-positive cells with mean levels of up to 40% for citrulline-specific and 54% for influenza-specific populations (see Additional file 1: Figure S3b) while for the general CD4+ population it was only around 10%. In the case of CXCR3 we saw strong signals amongst the influenza-specific T cells in some subjects, but generally only few of the citrulline-specific T cells were CXCR3+. Lastly, CCR6-expressing T cells were found to be rare, also amongst the influenza-reactive T cells and virtually absent amongst citrulline-specific T cells in the patients responding to treatment at the 6 months follow-up (see Additional file 1: Figure S3b).

Among all citrulline-specific cells we observed a number of cells co-expressing two or three of the chemokine receptors. Here it was most common to find CXCR5 in combination with either CXCR3 or CCR6 (data now shown). In contrast to this we found only a few influenza-specific cells co-expressing several chemokine receptors, these were mostly CXCR3+ cells carrying CCR6 and/or CXCR5 (data not shown).

## Discussion

With this study we demonstrate the utility of a multi-HLA class II tetramer approach to allow the unambiguous and simultaneous detection of rare self-reactive T cells in patients with seropositive arthralgia, i.e. individuals at risk of RA development, and those with early, untreated RA at the time of diagnosis. This technology could be useful in several settings where precise immune-monitoring would be beneficial, including longitudinal screening in the context of the stepwise development of RA, as well as follow-up studies in conjunction with therapy response or tolerance induction. This is demonstrated through the assessment of frequency and phenotype of citrulline-reactive T cells in RA patients at time of diagnosis and after 6 months of methotrexate therapy. Here, the numbers of citrulline-reactive T cells were diminished and the phenotype of certain specificities, but not others, was changed in patients responding to therapy and reaching minimal disease activity (DAS28 < 2.6).

Self-reactive CD4+ T cells are known to be rare, and several autoimmune diseases can be initiated through recognition of multiple target antigens. This is true e.g. for type 1 diabetes (T1D) and multiple sclerosis [23–25], but even more so for RA where common posttranslational modifications, like citrullination and carbamylation, are the target of the aberrant immune response [26, 27]. Clinical immune-monitoring of autoreactive CD4+ T cells in this setting will hence not only depend on sensitive methods to capture these rare events but also on the possibility to determine multiple specificities in the same assay. Multi-parameter flow cytometry is well-suited for these challenges and we here performed a first proof of principle study building on previous work in the setting of influenza vaccines [16]. We built a panel focusing on eight citrullinated T cell epitopes from four different proteins, that have previously been shown to be presented on HLA-DRB1*04:01 in the context of RA [5, 11, 14], and also included two viral control peptides [20, 21] (Table 1). For the phenotypic markers we first cover subdivisions of naïve versus central and effector memory cell states. In this context, HLA-tetramer studies from e.g. T1D have demonstrated that glutamic acid decarboxylase (GAD)-specific T cells are mainly of memory phenotype [28, 29]. Secondly, we included markers for the main signature T helper cell subsets including Th1 (CXCR3), Th17 (CCR6), Tfh (CXCR5), all of which have been postulated as interesting in the context of RA [30–35]. In fact, when examining the expression of these chemokine receptors on the citrulline-specific T cells we found the cells to be double- or even triple-positive for these markers in several instances, which also conformed with our observations for cells from the general CD4+ cell population.

For general validation of our assay we started out staining PBMC from healthy controls making use of the fact that autoreactive T cells, albeit in low numbers, form part of the normal T cell repertoire [5, 36–41]. As expected, the frequencies of citrulline-specific T cells were low but stably detectable in repeat experiments performed on samples from the same donors. Thus, besides the sensitivity in detecting these rare autoreactive T cells, we also demonstrated its robustness by replicating the stainings. To further corroborate these results, we applied our approach to repeat blood samples – three per patient – taken from 14 RA patients over the time course of 4–6 weeks. As all patients had stable long-standing disease we expected the autoreactive CD4+ T cell responses to be stable over this period of time. This was the case for around half of the patients for which we detected citrulline-specific T cells at all three time points. When investigating the differentiation state of the tetramer-positive cells we decided to focus on CD45RA and CCR7 to discriminate defined memory phenotypes from naïve T cell subsets and also included an activation marker besides the above-mentioned chemokine receptors. In contrast to influenza-specific T cells that were mainly found in effector and central memory T cell subsets, most citrulline-specific T cells in these quiescent RA patients belonged to the naïve or central memory compartment. However, interestingly, there is increasing evidence that the naïve T cell compartment is not homogeneous and can also include memory T cells such as the so called stem cell memory T cells, Tscm [42]. In our study, we defined naïve T cells as CD45RA and CCR7 double-expressing cells and did not include further markers to subcategorise such Tscm cells. It is nonetheless possible that said cells exist also in the autoreactive T cell repertoire in RA and explain the large proportion of citrulline-reactive T cells we detect in the naïve compartment.

The above-mentioned ex vivo staining approach relies on the availability of viable cells in the magnitude of around 20 million per time point. Still, in a clinical setting some samples would never reach such numbers. Small needle biopsies represent such scarce, but clinically interesting material. We had the opportunity to utilise samples of the Amsterdam LN biopsy cohort for our study [43], and in this context used cells that had been in vitro propagated prior to cryopreservation and tetramer staining. Nevertheless, we managed to find citrulline-reactive T cells of different specificities in all the RA patients and in many of the arthralgia patients. We also included some LN-relevant phenotypic markers, focusing on PD-1, CXCR5 as well as ICOS, to query the presence of a classical follicular T helper phenotype [44]. The phenotypic data was, probably due to the extended in vitro culture, inconclusive; nonetheless for some individuals we still detected prominent PD-1 positive populations (data not shown). Importantly, the protocol allows easy adjustment of the panel to capture the most relevant phenotypic markers based on the compartment from which the cells originate. So when e.g. studying RA synovial fluid, a different set of markers would be interesting to stain for compared to peripheral blood. An interesting marker would in that case be PD-1 that has recently been shown to characterise a specific subset of T helper cells in synovial fluid, the peripheral T helper cells, that in contrast to T follicular helper cells do not express CXCR5 and target inflamed tissues which is why they are close to absent in the periphery [45].

To further explore the capacity of our multi-tetramer panel in detecting differences over time, we utilised samples from early RA patients [19] which included peripheral blood obtained at time of RA diagnosis, prior to treatment initiations, and the subsequent 6 months follow-up visit. This gave us the possibility to ex vivo study untreated early RA patients and their T cell reactivities as well as the phenotype of these cells. We found for instance that three out of the four patients achieving remission by anti-rheumatic treatment had lower frequencies of citrulline-specific T cells at the follow-up. Notably, patient 5, for whom we also detected a decline in the frequency of citrulline-reactive CD4+ T cells at follow-up, could due to a missing DAS28-value at 6 months not be classified as achieving remission. Nevertheless, we suggest based on low DAS28-values at 3 and 13 months (2.24 and 2.53, respectively) and on the fact that no change in therapy was done at the 6 months follow-up visit that this patient probably had low disease activity or even an established remission. We also demonstrate the benefit of having different HLA class II tetramers in various channels in order to make specific sub-analyses here. Like this, we could detect a decrease in the frequency of memory T cells among α-enolase-and CILP/fibrinogen-specific T cells, but not in vimentin-reactive T cells. Generally, it has been difficult to predict clinical response in RA patients with both synthetic and biological DMARDs [46–54], and our small study implies that clinical response may parallel or result from the restriction of the autoimmune component of the disease.

## Conclusions

In our present study, we have used a multi-tetramer approach to demonstrate citrulline-reactive T cells in RA patients. Still, it should be noted that we consider the assay applicable to any disease restricted by HLA where there are a number of antigens involved or even when screening for T cells specific for different peptides of the same antigen. Other disease settings could for example be T1D with its restriction to both HLA-DR and -DQ loci [55] and the numerous autoantigens involved, like GAD and 60 kDa heat shock protein (HSP60) [56, 57] as well as coeliac disease with the HLA-DQ2 and -DQ8-restricted gluten-derived peptides [58, 59]. Importantly, we have addressed a number of candidate autoantigens in RA, but consider this only the beginning. As the disease starts developing many years prior to clinical onset and is likely to affect many organs, additional antigens are presumably involved in the disease. More studies are needed in order to get an overview of which specificities are important in each step of disease development and also whether it is possible to distinguish which T cells are public, i.e. present in many or the majority of patients, and which are private. Such information would be invaluable for further refinement of an immune-monitoring protocol and for designing future immunotherapies based on the exciting progress being made in RA in this regard [60, 61].

## Methods

### Patients and cell samples

All subjects (healthy donors as well as RA patients) were included based on their genetic profile by carrying one or two HLA-DRB1*04:01 alleles known to be associated with increased risk for rheumatoid arthritis. Fourteen ACPA-positive RA patients (men, %: 29), recruited at the Rheumatology clinic at the Karolinska University Hospital, were asked to participate in a longitudinal validation cohort and donate peripheral blood at three time points with intervals of 2–3 weeks (see Additional file 1: Table S1.1). Moreover, we had the opportunity to use baseline and 6 months follow-up PBMC samples from ten HLA-DRB1*04:01-positive patients (median age and range at inclusion, years: 43 (26–71); men, %: 40) enrolled in the LURA study at Karolinska University Hospital [19]. General characteristics for the selected newly diagnosed early RA patients, including antibody status, DAS28 at baseline and follow-up as well as medication after baseline sampling are displayed in Table 2. Seven HLA-DRB1*04:01-positive control subjects were recruited at the Uppsala Bioresource. PBMC, obtained from heparinised blood, were prepared by centrifugation over Ficoll-Hypaque gradients (GE Healthcare). The samples were cryopreserved in liquid nitrogen in 10% DMSO and 90% heat-inactivated fetal bovine serum.

Lymphoid tissue samples were derived from individuals undergoing ultrasound-guided inguinal LN core needle biopsy [41] recruited at the Academic Medical Center, Amsterdam, Netherlands (median age and range at inclusion, years: 50 (19–81); men, %: 17). Table S2 (Additional file 1) shows the demographics of the 12 included subjects that were selected based on the presence of at least one HLA-DRB1*04:01 allele. The general study cohort consists of individuals with arthralgia (joint pain without inflammation) and elevated levels of IgM-RF and/or ACPA as well as early arthritis patients with a disease duration ≤6 months and naïve to treatment with biologics. Besides this, healthy controls without any joint problems and RA-specific antibodies were included. Of the five arthralgia patients included in this study none developed RA within a follow-up period of 20–28 months after initial biopsy sampling.

### Production of HLA-DRB1*04:01 tetramers

HLA-DRB1*04:01 protein used within this study was recombinantly produced in S2 insect cells as previously described [20]. All peptides were synthesised at > 95% purity by GenScript (Piscataway, NJ, USA). The biotinylated monomer was loaded with the different peptides (Table 1) by incubation in the presence of n-octyl-β-D-glucopyranoside and Pefabloc SC (Sigma-Aldrich) and subsequently tetramerised using streptavidin conjugated to PE, PE-Cy5, PE-CF594 (BD Biosciences) or APC (Biolegend), respectively. Every single tetramer was loaded with one specific peptide during this individual assembly, so we ended up having ten different tetramers. Three of these were conjugated to PE and PE-CF594, respectively and two conjugated to APC and PE-Cy5, respectively (Table 1).

### Ex vivo detection of T cells by HLA class II tetramers

PBMC samples from RA patients were stained as previously described [16] using the tetramers loaded with the peptides according to Table 1 and subsequently labelled with anti-CD14 (MΦP9), anti-CD19 (4G7), Annexin V, anti-CD4 (SK3), anti-CD45RA (HI100), anti-CD25 (2A3), anti-CCR7 (3D12), anti-CCR6 (11A9), anti-CXCR3 (1C6), anti-CXCR5 (J252D4) antibodies. All of these antibodies were ordered from BD Biosciences, apart from the anti-CXCR5 antibody, which was purchased from Biolegend.

Between 8 and 40 million cells (median: 34.4 million) were used for staining for the longitudinal and early RA patient samples, respectively. The inclusion of the more specific markers, like CD25 and the different chemokine receptors in the staining panel allowed us an in-depth characterisation of the tetramer-positive cells from the samples. The early RA samples were furthermore labelled with anti-CD38 (HIT2, BD Biosciences) antibodies. Additional inclusion of an anti-CD3 antibody is recommendable, as it will facilitate gating of a pure CD4+ T lymphocyte population and moreover allow gating on CD4- T cells to check for general background staining by the tetramer. Samples were then acquired on a BD LSRFortessa flow cytometer and data was analysed using FlowJo, version 10.0.7 (Tree Star) and GraphPad Prism, version 6.0 h. The gating strategies for the different cohorts are presented in Figure S1 (Additional file 1). For calculating the frequency of antigen-specific cells we divided the total number of tetramer-positive cells in the bound fraction by the total number of CD4+ T cells. A cut-off of 1/10^6^ CD4+ T cells was applied when analysing the RA PBMC samples.

### Detection and sorting of lymph node T cells using HLA class II tetramers following in vitro cultures

As the number of cells that can be retrieved during a core needle biopsy is limited, we cultured the cells in vitro with PHA (2 μg/ml, Biochrom) and IL-2 (100 IU/ml, Sanquin) to specifically promote T cell activation and proliferation. After 5 days in culture the cells were counted and cryopreserved in liquid nitrogen. The expanded cells were stained directly after thawing using the eight citrulline-specific tetramers listed in Table 1 and incubated for 1 h at 37 °C. Thereafter, the cells were labelled with anti-CD14, anti-CD19, Annexin V, anti-CD4, anti-PD-1 (EH12), anti-CXCR5 and some (*n* = 7) also with anti-ICOS (DX29) antibodies. Both anti-PD-1 and anti-ICOS antibodies were ordered from BD Biosciences. Tetramer-positive cells were subsequently sorted on a BD influx cell sorter using the gating and sorting strategy shown in Figure S1b (Additional file 1). Cut-off for positivity for the different tetramer-cocktails were set to MFI > 400 for cit vim, MFI > 600 for cit α-eno and MFI > 320 for cit CILP/FGB. Additionally, the distribution of tentatively positive cells was taken into consideration when determining which samples were evaluated to be tetramer-positive.

### Statistics

Statistical analysis was performed when applicable and if so, is indicated in the respective figure legends. Comparing the frequency of tetramer-positive cells at baseline with the follow-up time point in early RA patients we applied Mann-Whitney test. *p*-values less than 0.05 were considered significant and marked with an asterisk.

## Supplementary information

**Additional file 1.** Supplementary information.

## Data Availability

The data that support the findings of this study are available from the corresponding author upon reasonable request.

## Abbreviations

RA: Rheumatoid arthritis
CILP: Cartilage intermediate layer protein
ACPA: Anti-citrullinated protein antibody
PBMC: Peripheral blood mononuclear cells
FGB: Fibrinogen beta chain
LN: Lymph node
LURA: Lung investigation in early RA patients
DAS: Disease activity score
RF: Rheumatoid factor
HA: Hemagglutinin
MP: Matrix protein
PHA: Phytohemagglutinin
IL-2: Interleukin-2
Tcm: Central memory T cell
Tem: Effector memory T cell
Temra: Terminal effector memory T cell
MFI: Mean fluorescence intensity
DMARDs: Disease-modifying anti-rheumatic drugs
GC: Glucocorticoids
T1D: Type 1 diabetes
Tscm: Stem cell memory T cell
GAD: Glutamic acid decarboxylase (GAD)
HSP60: 60 kDa heat shock protein
