# Supplementary material for: Multi-HLA class II tetramer analyses of citrulline-reactive T cells and early treatment response in rheumatoid arthritis
Source: BMC Immunol. 2020 May 18;21:27. doi: 10.1186/s12865-020-00357-w (PMC7236297; doi:10.1186/s12865-020-00357-w)
Supplement: Supplementary file 1 — Additional file 1. Supplementary information. [file 12865_2020_357_MOESM1_ESM.pptx]

## Slide 1
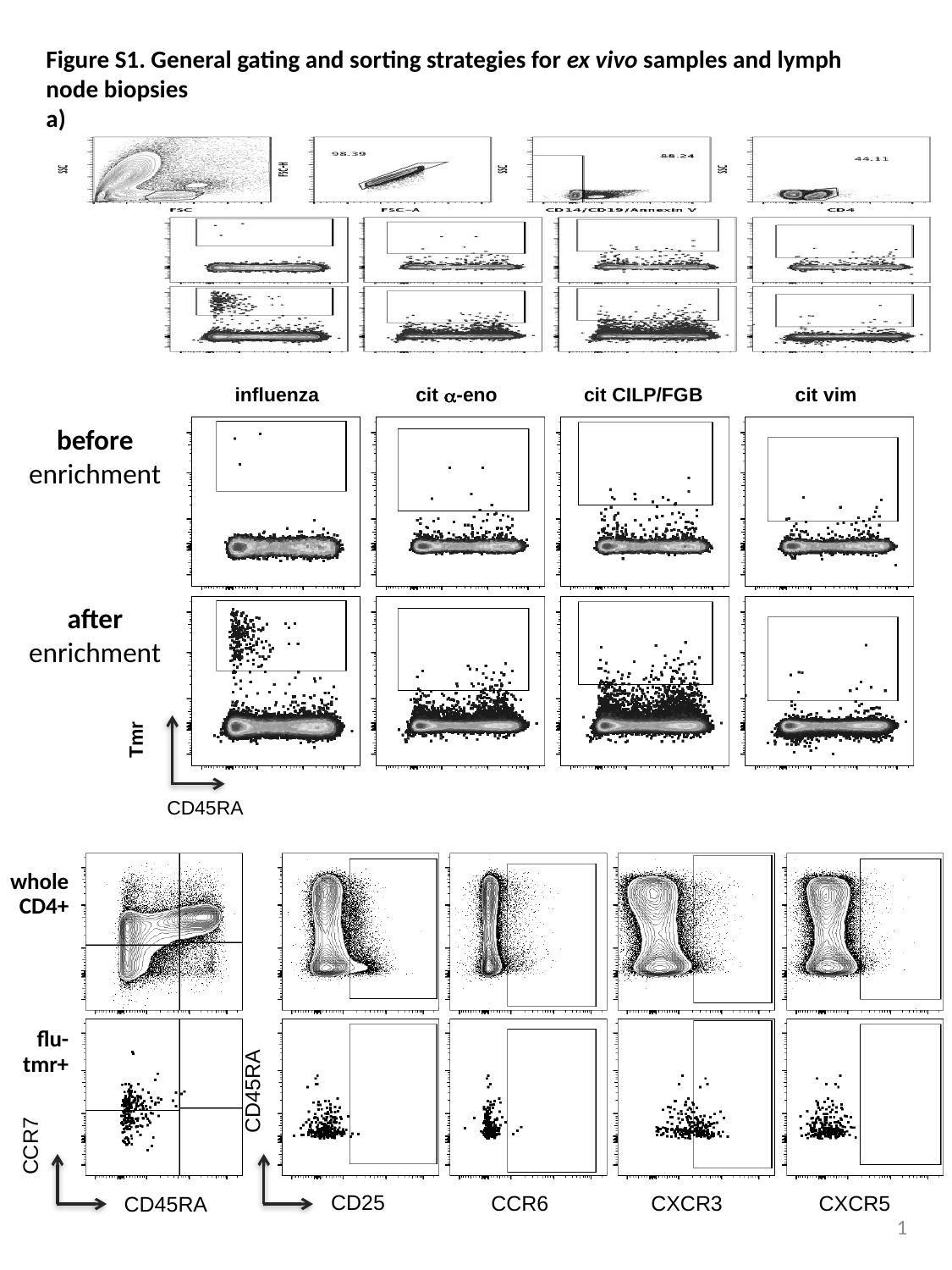

Figure S1. General gating and sorting strategies for ex vivo samples and lymph node biopsies
a)
influenza
cit a-eno
cit CILP/FGB
cit vim
before
enrichment
after
enrichment
Tmr
CD45RA
whole
 CD4+
flu-
tmr+
CD45RA
CCR7
 CD25
 CCR6
 CXCR3
 CXCR5
CD45RA
1

## Slide 2
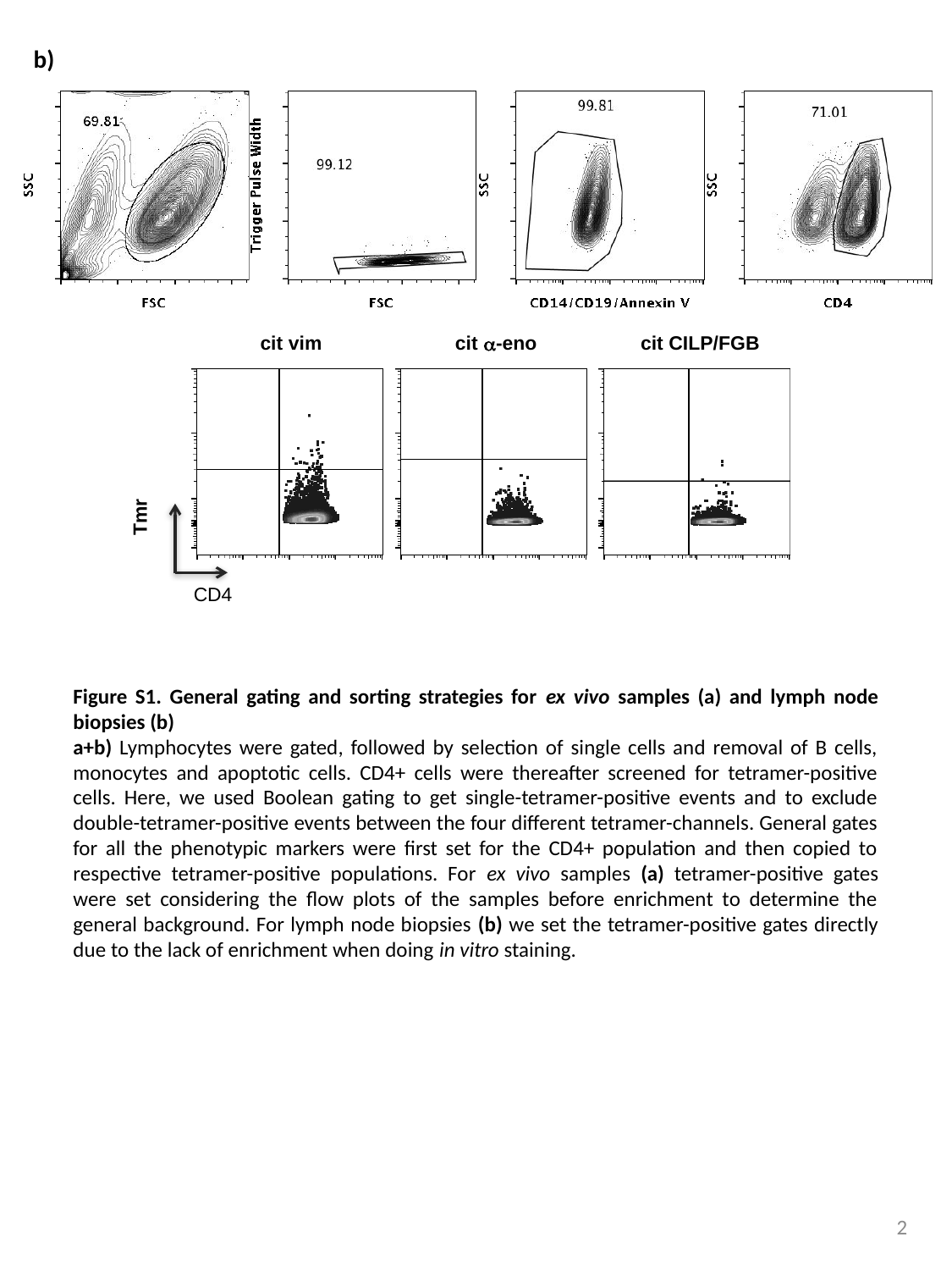

b)
cit vim
cit a-eno
cit CILP/FGB
Tmr
CD4
Figure S1. General gating and sorting strategies for ex vivo samples (a) and lymph node biopsies (b)
a+b) Lymphocytes were gated, followed by selection of single cells and removal of B cells, monocytes and apoptotic cells. CD4+ cells were thereafter screened for tetramer-positive cells. Here, we used Boolean gating to get single-tetramer-positive events and to exclude double-tetramer-positive events between the four different tetramer-channels. General gates for all the phenotypic markers were first set for the CD4+ population and then copied to respective tetramer-positive populations. For ex vivo samples (a) tetramer-positive gates were set considering the flow plots of the samples before enrichment to determine the general background. For lymph node biopsies (b) we set the tetramer-positive gates directly due to the lack of enrichment when doing in vitro staining.
2

## Slide 3
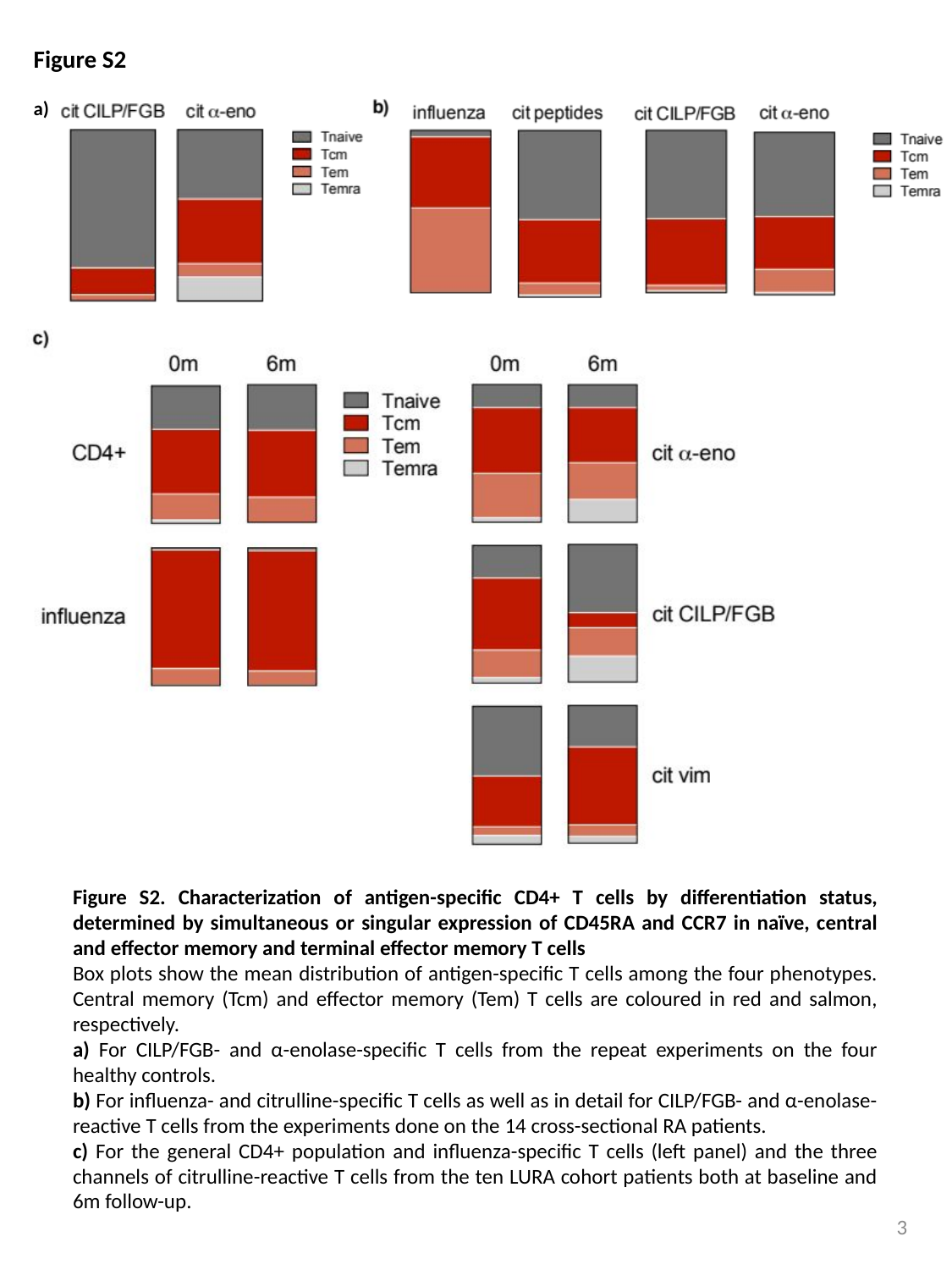

Figure S2
a)
Figure S2. Characterization of antigen-specific CD4+ T cells by differentiation status, determined by simultaneous or singular expression of CD45RA and CCR7 in naïve, central and effector memory and terminal effector memory T cells
Box plots show the mean distribution of antigen-specific T cells among the four phenotypes. Central memory (Tcm) and effector memory (Tem) T cells are coloured in red and salmon, respectively.
a) For CILP/FGB- and α-enolase-specific T cells from the repeat experiments on the four healthy controls.
b) For influenza- and citrulline-specific T cells as well as in detail for CILP/FGB- and α-enolase-reactive T cells from the experiments done on the 14 cross-sectional RA patients.
c) For the general CD4+ population and influenza-specific T cells (left panel) and the three channels of citrulline-reactive T cells from the ten LURA cohort patients both at baseline and 6m follow-up.
3

## Slide 4
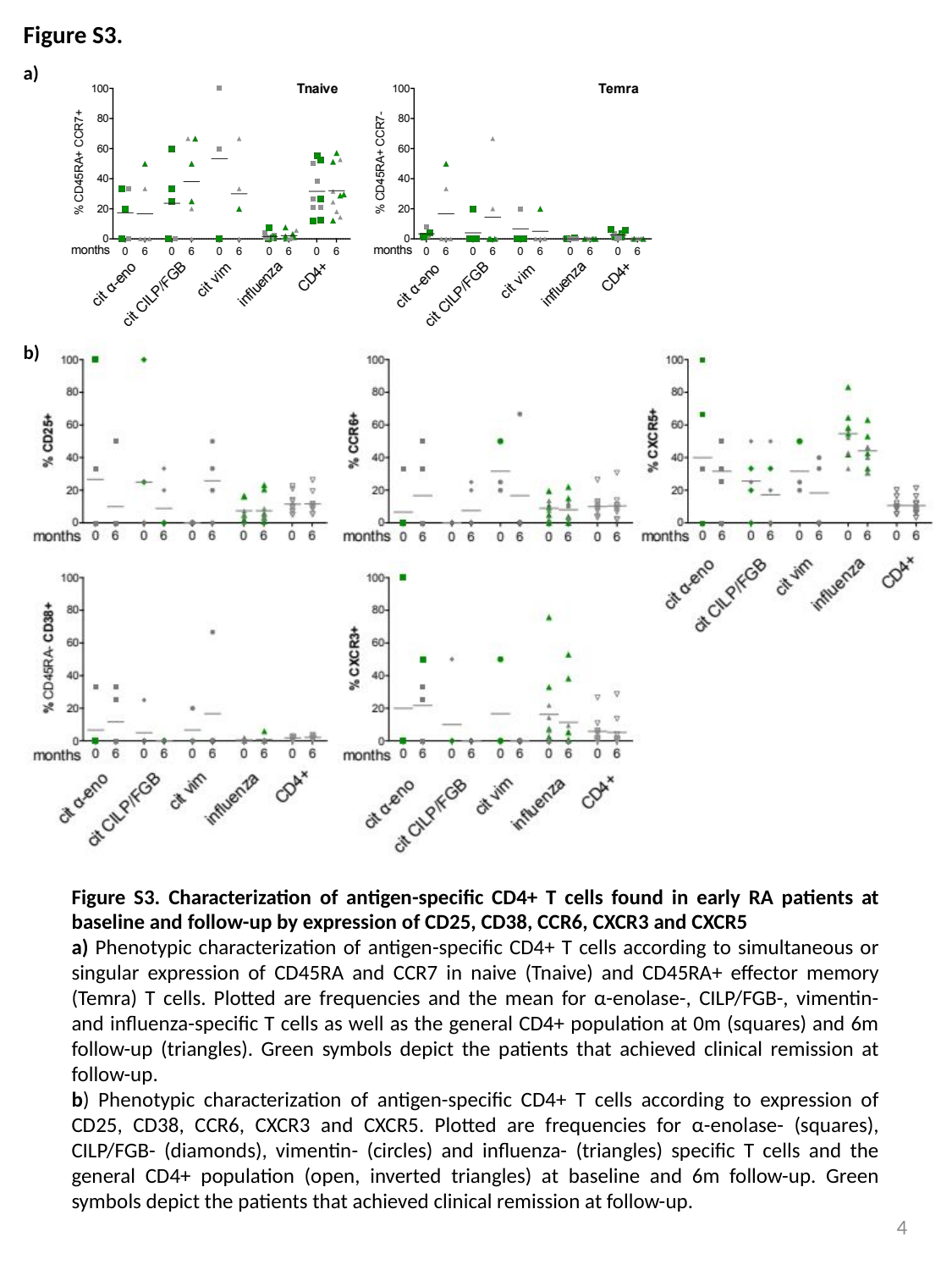

Figure S3.
a)
b)
Figure S3. Characterization of antigen-specific CD4+ T cells found in early RA patients at baseline and follow-up by expression of CD25, CD38, CCR6, CXCR3 and CXCR5
a) Phenotypic characterization of antigen-specific CD4+ T cells according to simultaneous or singular expression of CD45RA and CCR7 in naive (Tnaive) and CD45RA+ effector memory (Temra) T cells. Plotted are frequencies and the mean for α-enolase-, CILP/FGB-, vimentin- and influenza-specific T cells as well as the general CD4+ population at 0m (squares) and 6m follow-up (triangles). Green symbols depict the patients that achieved clinical remission at follow-up.
b) Phenotypic characterization of antigen-specific CD4+ T cells according to expression of CD25, CD38, CCR6, CXCR3 and CXCR5. Plotted are frequencies for α-enolase- (squares), CILP/FGB- (diamonds), vimentin- (circles) and influenza- (triangles) specific T cells and the general CD4+ population (open, inverted triangles) at baseline and 6m follow-up. Green symbols depict the patients that achieved clinical remission at follow-up.
4

## Slide 5
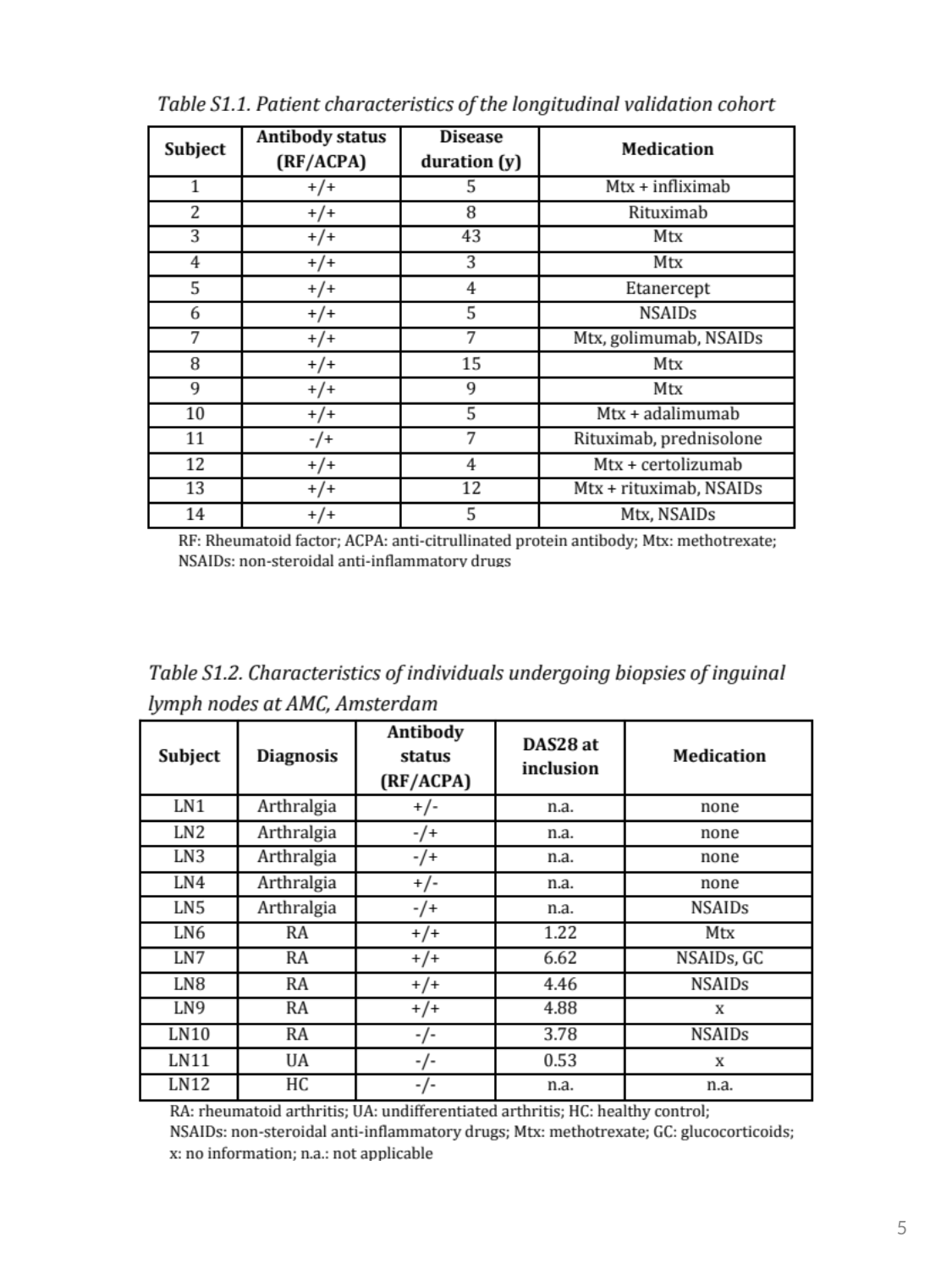

5
